# COVID-19 infection among pregnant and non-pregnant women: Comparison of biochemical markers and outcomes during COVID-19 pandemic, A retrospective cohort study

**DOI:** 10.1016/j.amsu.2022.103527

**Published:** 2022-03-28

**Authors:** Muhammad Sohaib Asghar, Muhammad Ali Siddiqui, Sadia Iqbal, Avinash, Muhammad Junaid Tahir, Farah Yasmin, Najeebullah Chughtai, Farmanullah Khan, Tooba Ahmed Kirmani, Iqra Lareeb

**Affiliations:** a Department of Internal Medicine, Dow University of Health Sciences–Ojha Campus, Karachi, Pakistan; b Department of Internal Medicine, Liaquat National Hospital & Medical College, Karachi, Pakistan; c Department of General Surgery, Liaquat National Hospital & Medical College, Karachi, Pakistan; d Department of Internal Medicine, Lahore General Hospital, Lahore, Pakistan; e Department of Internal Medicine, Dow Medical College, Dow University of Health Sciences, Karachi, Pakistan

**Keywords:** Pandemic, SARS-CoV-2, Neonatal, Maternal, Pregnancy

## Abstract

**Background:**

& Objectives: We conducted this single-centered retrospective study including female patients infected with COVID-19 with aim to compare laboratory findings and the outcomes between pregnant and non-pregnant women infected with COVID-19. Previous data rendered pregnant women as vulnerable population for COVID-19**.**

**Methods:**

We included 131 patients in our analysis out of which 60 were pregnant females and rest 71 were non-pregnant females.

**Results:**

Factors like fatigue, total leukocyte count (TLC) and neutrophils were higher in pregnant patients, while mean age, fever, hemoglobin, ferritin, D-dimer and use of mechanical ventilation was lower in pregnant patients as compared to non-pregnant females.

**Conclusion:**

Our study concluded that COVID-19 do not show significant high risk of disease severity when compared with non-pregnant females of similar age group.

## Highlights

• Previous studies reported that pregnant women are vulnerable population appearing to be at higher risk of morbidity and mortality from COVID-19.
• Our study concluded that COVID-19 do not show significant high risk of disease severity in pregnancy when compared with non-pregnant females of similar age group.

## 1 Introduction

A recent article on the effect of severe acute respiratory coronavirus 2 (SARS-CoV-2) infection in pregnancy on maternal and neonatal outcomes in Africa, highlighted the fact that data on COVID-19 in pregnancy and its effects on maternal and neonatal health are limited [1]. SARS-CoV-2 is a deadly virus that resulted in coronavirus disease 2019 (COVID-19). It started in Wuhan (China), in December 2019; presently a well-recognized pandemic all around the globe [2]. Severe maternal morbidity resulting from COVID-19 and perinatal deaths have been reported but still there are limited case series reporting on impact of COVID-19 on women during pregnancy [3]. Data that is available on COVID-19 in pregnant women suggests that they may experience symptoms indistinguishable from those of the general population and also the vertical transmission from mother to child is unlikely [4].

The women during pregnancy undergoes many physiological changes, amongst all most highlighted is alterations in immunity and hormones which ensure viability and development of a fetus, in presence of a combative maternal immune system [5,6]. Thus, increasing the susceptibility of a pregnant woman to severe infections [5]. Moreover, the individuals who have mild COVID-19 symptoms may also be at increased risk of severe pneumonia and poor pregnancy consequences, specifically those involving complications such as pre-eclampsia [5].

Importantly, the shared need for better pregnancy outcomes in pregnant women with COVID-19 sets the ground for further research on this topic. Therefore, this study aims to compare laboratory findings (mainly hematological and inflammatory markers) and the outcomes of pregnant women as compared to non-pregnant women infected with COVID-19. It was rendered in the previous study that pregnant women are vulnerable population appearing to be at higher risk of morbidity and mortality from COVID-19 than age-matched, nonpregnant women [1]. We aim to determine such outcomes in our population.

## 2 Methodology

We conducted this single-centered retrospective study including female patients infected with COVID-19 with aim to compare laboratory findings and the outcomes between pregnant and non-pregnant women infected with COVID-19.

The study ensured that the privacy of each patient was adequately protected. A study protocol was approved by the ethical review committee and registered with a national registry (UIN# LNH/ERC/07/2021/73) from the institutional reviewer board of Liaquat National Hospital. The study was carried out in accordance with the Helsinki Declaration and STROCSS criteria [7]. Informed written consent was waived owing to the retrospective nature of this study. During screening for the disease, authors back-to-back assessed all the possible eligible cases for compliance with the inclusion criteria for the study. The clinical characteristics and laboratory test results of patients were extracted from electronic medical records and reviewed by the designated authors.

There were two study groups, one having pregnancy while infected with the virus and the other group also infected with COVID-19 in a reproductive age group but not pregnant at the time. COVID-19 positivity was detected via either nasopharyngeal or oropharyngeal swab for PCR. The diagnostic kit used exploits the principle of real-time fluorescence (RT-PCR), USA-WA1/2020 stock concentration 2.8E+05 TCID50/mL, with a lower detection limit of 0.003 TCID50/mL. A total of 131 patients met the inclusion criteria out of which 60 were pregnant females and rest 71 were non-pregnant females.

Statistical analyses were performed using the statistical software Statistical Package for Social Sciences (SPSS) version 25.0 (Armonk, NY, USA). Basic statistical analyses including means, standard deviations, frequencies, percentages, etc., were computed. Comparisons between pregnant and nonpregnant groups were made using the Chi-square test or Fisher exact test for categorical variables, whereas the student *t*-test was used for continuous variables. A *p*-value of <0.05 was considered statistically significant.

## 3 Results

The mean age of pregnant females was much lower than non-pregnant females in our study (p = 0.003) while mean body mass index (BMI) was indifferent (p = 0.082). Fatigue (78.3%) was the most predominant symptom in pregnant patients (p = 0.043) followed by cough (68.3%) and dyspnea (66.7%), while fever (84.5%) was predominantly a feature of non-pregnant COVID-19 patients (p = 0.003) as shown in Table 1. Laboratory markers most likely to differ among the groups were lower hemoglobin (p < 0.001), serum ferritin (p = 0.001) and D-dimer (p = 0.040) in pregnant patients while a higher TLC (p = 0.033) and neutrophil count (p = 0.041) was also noted in pregnant females as shown in Table 2.

**Table 1:** Baseline characteristics of the study population (n = 131).

Characteristics	Pregnant (n = 60)	Non-pregnant (n = 71)	P-value
Mean age (in years)	28.11 ± 5.24	31.65 ± 7.97	0.003*
Mean BMI (kg/m^2^)	27.92 ± 3.21	29.35 ± 5.59	0.082*
Mean gestational age (in week)	31.46 ± 6.13	–	–
Parity	1.78 ± 1.33	–	–
Gravidity	3.27 ± 2.04	–	–
Nulliparous	11 (18.3%)	–	–
Presence of comorbidities	7 (11.6%)	13 (18.3%)	0.292**
Fever	37 (61.7%)	60 (84.5%)	0.003**
Cough	41 (68.3%)	56 (78.9%)	0.170**
Dyspnea	40 (66.7%)	53 (74.6%)	0.316**
Fatigue	47 (78.3%)	44 (62.0%)	0.043**
Gastrointestinal symptoms	19 (31.7%)	18 (25.4%)	0.424**

Data presented as mean ± standard deviation or frequency (percentage).

P-Value calculated by * student's t-test ** Chi-square test.

BMI: body mass index; n: number of subjects.

**Table 2:** Mean laboratory parameters at admission among the study groups (n = 131).

#	Laboratory parameters	Pregnant (n = 60)	Non-pregnant (n = 71)	p-value
1	Hemoglobin (<9.0 g/dL)	9.60 ± 2.38	11.09 ± 2.14	<**0.001**^
		21 (35.0%)	7 (9.9%)	<0.001*
2	Platelets (<150 × 10^9^/L)	213.00 ± 85.71	242.04 ± 115.50	0.110^^^
		3 (5.0%)	4 (5.6%)	1.000**
3	TLC (>11.0 × 10^9^/L)	11.64 ± 5.19	9.87 ± 4.15	**0.033**^^^
		26 (43.3%)	25 (35.2%)	0.342*
4	Neutrophils (>75%)	74.88 ± 8.35	71.36 ± 11.17	**0.041**^^^
		26 (43.3%)	28 (39.4%)	0.652*
5	Lymphocytes (<20%)	19.40 ± 7.75	22.29 ± 9.91	0.069^^^
		18 (30.0%)	17 (23.9%)	0.435*
6	CRP (>10 mg/L)	75.95 ± 100.80	100.74 ± 99.35	0.160^^^
		49 (81.7%)	61 (85.9%)	0.509*
7	LDH (>250 U/L)	549.20 ± 284.91	526.30 ± 234.39	0.615^^^
		51 (85.0%)	64 (90.1%)	0.371*
8	Ferritin (>250 ng/mL)	149.12 ± 138.05	866.31 ± 1670.27	**0.001**^^^
		15 (25.0%)	46 (64.8%)	<0.001*
9	Procalcitonin (>0.50 ng/mL)	1.08 ± 2.16	2.47 ± 6.42	0.112^^^
		11 (18.3%)	24 (33.8%)	0.046*
10	D-dimer (>0.50 mcg/mL)	3.97 ± 4.75	7.40 ± 12.01	**0.040**^^^
		58 (96.7%)	63 (88.7%)	0.108**

Data presented as mean ± standard deviation or frequency (percentage).

^^^ P-Value calculated by student's t-test, * Chi-square test, ** Fisher's exact test.

TLC: total leukocyte count; CRP: c-reactive protein; LDH: lactate dehydrogenase; n: number of subjects.

Length of hospital stay was higher in non-pregnant females (p = 0.009), while other disease outcomes like intubation, bilateral pulmonary infiltrates, development of acute respiratory distress syndrome (ARDS), and thrombotic events was statistically indifferent among the groups as shown in Table 3. Only significant difference was lesser need for mechanical ventilation in pregnant patients (p = 0.047), while mortality rates were 5.0% in pregnant versus 15.5% in non-pregnant patients (p = 0.053). The pregnancy outcomes reported were vaginal delivery in 25%, Cesarean section in 70%, premature birth in 6%, still birth/intrauterine death (IUD) in 3% and preeclampsia was present in 8% of those pregnancies.

**Table 3:** Major outcomes of COVID-19 among the pregnant and non-pregnant population (n = 131).

#	Outcome variables	Pregnant (n = 60)	Non-pregnant (n = 71)	p-value
1	Length of hospital stay (in days)	6.63 ± 3.51	9.16 ± 6.60	0.009*
2	Mechanical ventilation (BiPAP)	7 (11.7%)	18 (25.4%)	0.047**
3	Intubated	2 (3.3%)	9 (12.7%)	0.055**
4	Bilateral pulmonary infiltrates	16 (26.7%)	27 (38.0%)	0.168**
5	ARDS	4 (6.7%)	8 (11.3%)	0.363**
6	Multi-organ failure (MODS)	5 (8.3%)	12 (16.9%)	0.098**
7	Thrombotic events	3 (5.0%)	7 (9.9%)	0.297**
8	Mortality	3 (5.0%)	11 (15.5%)	0.053**
9	Vagina delivery	15 (25.0%)	–	–
10	C-section	42 (70.0%)	–	–
11	Premature birth	4 (6.6%)	–	–
12	Still birth/IUD	2 (3.3%)	–	–
13	Preeclampsia	5 (8.3%)	–	–

Data presented as mean ± standard deviation or frequency (percentage).

* P-Value calculated by student's t-test; ** P-Value calculated by Chi-square test.

ARDS: Acute respiratory distress syndrome; C-section: Caesarean section; IUD: intrauterine death; COVID-19: coronavirus disease 2019; n: number of subjects.

## 4 Discussion

Nachega et al. [1] in their study have focused on the outcomes of COVID-19 in pregnant females by factors such as immune changes leading to increased hospitalization, ICU stay, mechanical ventilation and mortality. But our study findings were contradicting their assessment as few of our pregnant infected with COVID-19 had severe outcomes as compared to non-pregnant females. A case series of 8 pregnant females showed a milder course of COVID-19 and recommended monitoring of TLC, lymphocyte count and CRP [8]. Cheng et al. [9] studied 31 pregnant females with COVID-19 in Wuhan and observed lesser disease severity and decreased incidence of dyspnea and asthenia when compared to non-pregnant females, with favorable neonatal outcomes. Despite that, higher TLC, neutrophil count and elevated D-dimer were observed in pregnant patients [9]. High TLC, neutrophil count and CRP were also reported to be reliable parameters in COVID-19 among pregnant patients in a review article [10]. Another study from Wuhan, China on pregnant females infected with COVID-19 had lesser symptomatology and decreased length of hospital stay when compared to non-pregnant females. However, TLC, neutrophil count, CRP, PCT and D‐dimer were more raised in pregnant women [11].

London et al. [12] in their study observed that symptomatic pregnant patients have a higher risk of preterm delivery and need of oxygen in COVID-19 infection. However, frequent studies have reported asymptomatic presentation of COVID-19 in pregnant females [8,9,11,12]. Data from Italy had 84% symptomatic pregnant patients among whom 18% exhibited severe disease [13]. Pre-term delivery was another important outcome in 12% of those, with higher BMI, fever and dyspnea found associated with disease severity [13]. Early data from Singapore also linked obesity and increased maternal age with disease severity [14]. Favorable neonatal outcomes were also reported in 118 pregnant patients from Wuhan, China apart from 14 preterm deliveries [15]. Elevated TLC was observed in 14%, lymphopenia in 44%, elevated D-dimer in 81%, raised LDH in 29%, PCT in 25% and CRP in 67% of those pregnant patients [15]. Initial high CRP and post-partum rising D-dimer were reported by multiple studies [8,14,16]. In contrast, our study reported substantially low CRP levels in the pregnant population as compared to non-pregnant COVID-19 infected subjects.

A case series of 5 pregnant females infected with COVID-19 showcased ground glass opacities as a predominant radiological finding [17]. Similar radiological findings were observed in another 7 pregnant patients from Wuhan [18]. Data from France of 100 pregnant COVID-19 infected females suggested higher BMI and lower lymphocyte counts were more likely factors for intensive care during hospitalization [19]. Another study compared pregnant females having COVID-19 infection, with those not infected with COVID-19 but similar characteristics like age, parity, number of pregnancies and comorbidities [20]. A significantly higher neutrophil count, CRP levels and lower lymphocyte counts were observed in pregnant COVID-19 infected females [20]. These parameters when analyzed with non-pregnant reproductive age females in our study results also showed higher neutrophil count and decreased lymphocytes in pregnant females, but CRP was significantly higher in non-pregnant patients infected with COVID-19. Lastly, a study on 16 pregnant COVID-19 patients from Morocco had favorable outcomes in their population [21].

The limitations of this study include a single institution analysis and a relatively small sample size. The study only included hospitalized COVID-19 patients hence might be subjected to selection bias. To conclude, our pregnant females did not report disease severity as much as the non-pregnant females of reproductive age group. However, the literature signifies the lethal virus can increase the mortality rates in pregnancy against those not infected with virus during pregnancy period.

## Ethical approval statement

Ethical approval was taken in this study from institutional review board (Ref:App.# LNH/ERC/07/2021/73), and consent to participate was not required due to retrospective nature of the study.

## Funding sources

No funding required for the study.

## Author contribution

M.A.S, M.S.A, and S.I conceived the idea, A, N.C, F.Y, and M.J.T retrieved the data, did write up of letter, and finally, T.A.K, I.L, and F.K reviewed and provided inputs. All authors approved the final version of the manuscript.

## Registration of research studies

Name of the registry: Liaquat National Hospital and Medical College.

Unique Identifying number or registration ID: LNH/ERC/07/2021/73.

Hyperlink to your specific registration (must be publicly accessible and will be checked):

## Guarantor

Muhammad Sohaib Asghar and Muhammad Junaid Tahir.

## Data availability statement

All data will be made available on a reasonable request to the corresponding author.

## Consent

Consent to participate was not required due to retrospective nature of the study.

## Provenance and peer review

Not commissioned, externally peer reviewed.

## Declaration of competing interest

The authors have no conflicts of interest to declare.
